# 1654. Impact of a Daily Web-Based Educational Quiz on Appropriateness of Pediatric Outpatient Antibiotic Prescribing

**DOI:** 10.1093/ofid/ofad500.1488

**Published:** 2023-11-27

**Authors:** Brittany J Lehrer, Ritu Banerjee, Sophie E Katz

**Affiliations:** Vanderbilt University Medical Center, Nashville, Tennessee; Vanderbilt University Medical Center, Nashville, Tennessee; Vanderbilt University Medical Center, Nashville, Tennessee

## Abstract

**Background:**

Nearly ⅓ of outpatient antibiotic prescriptions are unnecessary or inappropriate. QuizTime is a web-based application based on Test-Enhanced Learning Theory that delivers daily case-based questions to a learner’s email or cell phone. We performed a prospective cohort study to evaluate the impact of a QuizTime module on appropriateness of pediatric outpatient antibiotic prescriptions.

**Methods:**

Participants received one question daily for 10 days starting in July 2022. Quiz topics included age-appropriate antibiotic duration for acute otitis media (AOM), empiric antibiotics for uncomplicated urinary tract infections (UTI), and empiric antibiotics and duration for community-acquired pneumonia (CAP) (**Table 1**). Participants were pediatric prescribers (physician, resident, physician assistant, nurse practitioner) in a primary care, urgent care, retail health, or emergency department setting. Antibiotic prescription data were collected during the “baseline” period (Jul 1, 2021 – Jun 30, 2022) though 3 months after QuizTime participation (Jul 15 – Oct 15 2022) defined as the “post-quiz” period. Pre- and post-intervention data were analyzed in aggregate (not at the provider-level). Significance was determined by calculating a 95% confidence interval (CI) for the difference of proportions. Outcome measures are listed in **Table 1**.

**Figure 1.**
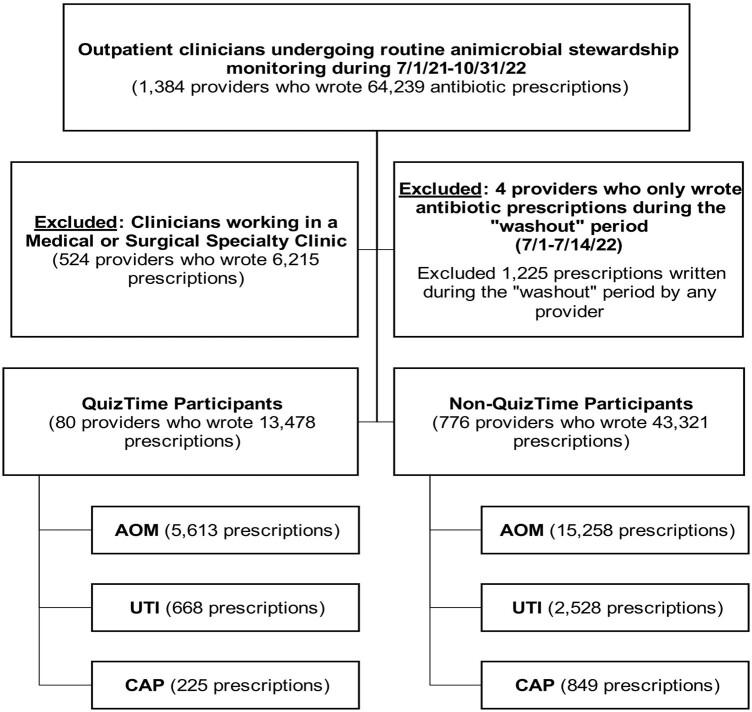
Prisma diagram showing providers and oral antibiotics prescribed in each cohort.

**Figure:**
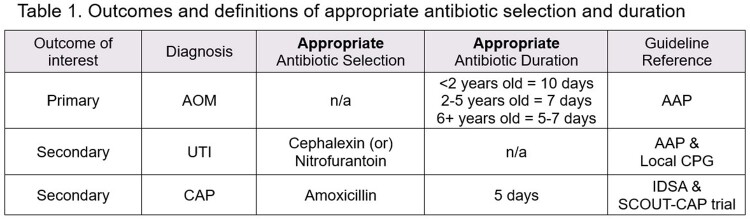

**Results:**

Eighty clinicians participated in QuizTime totaling 13,478 antibiotic prescriptions [AOM 5,613 (41.6%), UTI 668 (5.0%), CAP 225 (1.7%)] compared to 776 non-QuizTime clinicians totaling 43,321 antibiotic prescriptions [AOM 15,258 (35.2 %), UTI 2,528 (5.8%), CAP 849 (2.0%)] (**Figure 1**). AOM guideline-concordant antibiotic duration was significantly higher among participants than non-participants (**Table 2**). There were encouraging trends towards improvement in secondary outcomes (**Table 3, 4**).

**Figure:**
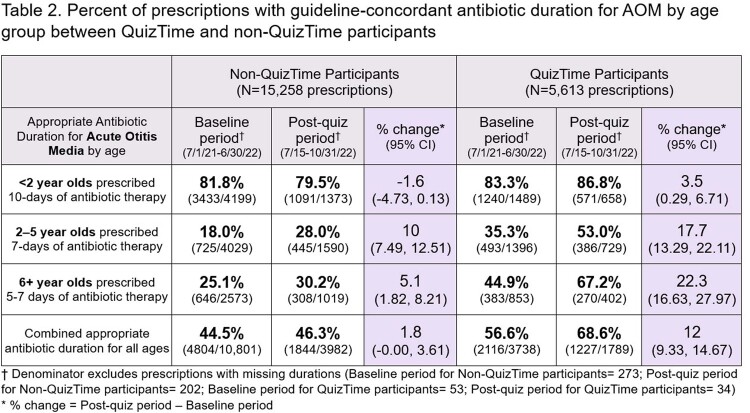

**Figure:**
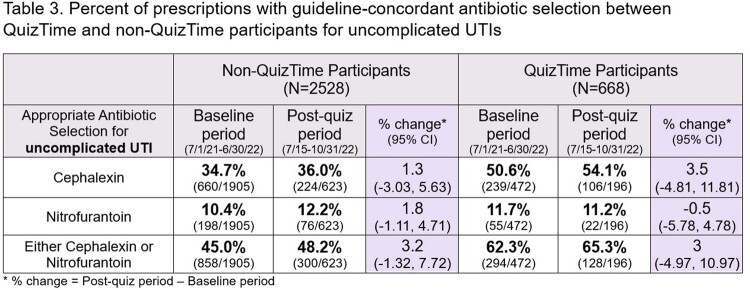

**Figure:**
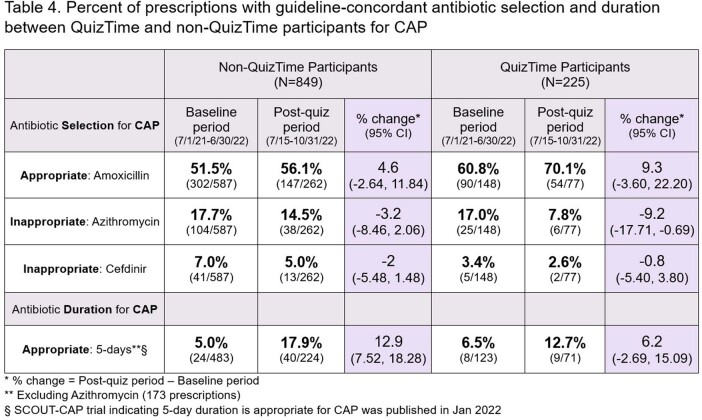

**Conclusion:**

Clinicians who participated in a QuizTime module on pediatric outpatient antibiotic prescribing showed improvement in antibiotic appropriateness suggesting web-based microlearning modules are an effective tool for disseminating antibiotic stewardship education. Creation, dissemination, and evaluation of additional QuizTime modules is ongoing.

**Disclosures:**

**Ritu Banerjee, MD, Ph.D**, bioMerieux: Grant/Research Support|bioMerieux: company is providing partial support for an ongoing trial unrelated to submitted abstract **Sophie E. Katz, MD MPH**, Dolly Parton Pediatric Infectious Diseases Research Funds: Grant/Research Support|Optum: Advisor/Consultant|Pfizer: Grant/Research Support

